# A Magnetic Actuator Device for Fully Automated Blinking in Total Bidirectional Eyelid Paralysis: First Proof of Concept in a Human Participant

**DOI:** 10.1167/tvst.13.5.2

**Published:** 2024-05-02

**Authors:** Kevin E. Houston, Manarshhjot Singh, Ashkan E. Sedeh, Eleftherios I. Paschalis

**Affiliations:** 1 Departments of Neurology and Ophthalmology, University of Massachusetts Chan Medical School, Worcester, MA, USA; 2 Department of Neurology, University of Massachusetts Chan Medical School, Worcester, MA, USA; 3 Department of Ophthalmology, Massachusetts Eye and Ear, Boston, MA, USA; 4 Schepens Eye Research Institute, Harvard Medical School, Boston, MA, USA

**Keywords:** prosthesis, blink reanimation, ptosis, rehabilitation

## Abstract

**Purpose:**

Currently, no solution exists to restore natural eyelid kinematics for patients with complete eyelid paralysis due to loss of function of both the levator palpebrae superioris and orbicularis oculi. These rare cases are prone to complications of chronic exposure keratopathy which may lead to corneal blindness. We hypothesized that magnetic force could be used to fully automate eyelid movement in these cases through the use of eyelid-attached magnets and a spectacle-mounted magnet driven by a programmable motor (motorized magnetic levator prosthesis [MMLP]).

**Methods:**

To test this hypothesis and establish proof of concept, we performed a finite element analysis (FEA) for a prototype MMLP to check the eyelid-opening force generated by the device and verified the results with experimental measurements in a volunteer with total bidirectional eyelid paralysis. The subject was then fitted with a prototype to check the performance of the device and its success.

**Results:**

With MMLP, eye opening was restored to near normal, and blinking was fully automated in close synchrony with the motor-driven polarity reversal, with full closure on the blink. The device was well tolerated, and the participant was pleased with the comfort and performance.

**Conclusions:**

FEA simulation results conformed to the experimentally observed trend, further supporting the proof of concept and design parameters. This is the first viable approach in human patients with proof of concept for complete reanimation of a bidirectionally paretic eyelid. Further study is warranted to refine the prototype and determine the feasibility and safety of prolonged use.

**Translational Relevance:**

This is first proof of concept for our device for total bidirectional eyelid paralysis.

## Introduction

Bidirectional eyelid paralysis, or complete eyelid paralysis, is a rare condition defined as the inability to naturally generate the forces needed to open or close the eye. This occurs when two distinct cranial nerve pathways are affected: cranial nerve III (oculomotor) and cranial nerve VII (facial). Data on the prevalence of this disorder are not available in the literature, but the clinical experience of the authors suggests that the condition is uncommon but often visually devastating due to chronic corneal exposure, which can lead to corneal blindness. Lack of eyelid movement affects corneal epithelial integrity, and, if left untreated, defects lead to stromal scarring, infection, and corneal melting. Injuries or disease may occur anywhere along the neural pathway from the brainstem nuclei (e.g., infarction),^1^ nerve tracts (e.g., trauma),^2^ neuromuscular junctions (myasthenia gravis),^3^^,^^4^ or musculature (e.g., chronic progressive external ophthalmoplegia, or inhabitant gene mutation in Kearns–Sayre syndrome).

Currently, there is no effective treatment to restore eyelid motility in bidirectional eyelid paralysis. Interventions mainly focus on mitigating corneal complications with protective scleral contact lenses, and in severe cases where corneal melting occurs a prosthetic cornea may be implanted (e.g., Boston Keratoprosthesis). The challenge is that techniques effective for simple unidirectional impairments (i.e., blepharoptosis and lagophthalmos^5^) rely on preservation of the antagonist neuromuscular system. For example, the frontalis sling procedure for severe blepharoptosis utilizes the frontalis muscle (VII) as a substitute for the paretic levator muscle (III); that is, the lid is opened by raising the brow. However, these procedures are futile in bidirectional eyelid paralysis, as the frontalis muscle is also dysfunctional. The preferred treatment for blepharoptosis consists of implanting a gold or titanium weight in the eyelid, thereby closing the lid by the weight of the implant; however, to restore eyelid motility, the levator (III) must be intact or the eye remains closed.

For eyelid paralysis, multiple eyelid actuation mechanisms have been proposed previously,^6^ some of which can be used for bidirectional eyelid paralysis. These include, but are not limited to, implantable lid loads,^7^ electrical stimulation,^8^ artificial muscle,^9^ a wearable soft actuator,^10^^–^^12^ variations of solenoid lid implants,^13^^–^^17^ and the magnetic levator prosthesis.^18^^–^^21^

The soft actuator, a prior device that the authors referred to as the *eyelid gating mechanism* (Supplementary Fig. S2), consists of a soft actuator placed on the upper eyelid. The soft actuator is activated by a servo motor fixed on a spectacle frame and a pulley system^12^ that acts at the corners of the soft actuator. Rotation of the servo motor deforms the soft actuator in a manner that mimics the natural blink of the eyelid. Electromyographic signals from the healthy side mimic the motion in the affected eye.^11^ This approach is suitable for complete paralysis because the eyelid motion can be simulated for both opening and closing the eye by simply reversing the direction of the servo motor. The major drawback is the presence of multiple mechanical components in motion that make the entire system bulky and slow. Additionally, with a physical connection between the spectacle frame and the wearable robot, patients would be susceptible to injury in the event of a direct blow or a fall and cannot remove the spectacle frame when they choose to.

A solenoid lid implant (Supplementary Fig. S3) design has also been considered previously for eyelid paralysis. In this approach, the authors suggested that a sling be sutured to the bottom edge of the upper eyelid and attached to a solenoid placed on the zygomatic arch. A blink is detected by the relaxation of the levator palpebrae superioris muscle, which discharges a capacitor to activate the solenoid. The solenoid pulls the sling to close the upper eyelid, and natural anatomy then reopens the eye upon solenoid release. Studies on rabbits^15^ and human cadavers^16^ confirmed that the blinking force necessary to close the eyelid can be achieved using this method. The system can address bidirectional eyelid paralysis by employing a double-acting solenoid. One reported drawback in the design pertains to the “free space” necessary for solenoid movement, which living tissue lacks naturally. Another disadvantage arises from the actuation direction not aligning with the natural eyelid opening and closing direction.

Another proposed design, the magnetic levator prosthesis (MLP) serves as a passive device for the non-invasive treatment of severe blepharoptosis.^18^ It is comprised of a magnetic array crafted from small permanent magnets embedded within biocompatible polydimethylsiloxane (PDMS). This magnetic array securely and reversibly attaches to the upper eyelid using standard hydrocolloid-based medical adhesive, such as Tegaderm (3M, St. Paul, MN). The magnetic eyelid array remains suspended within the magnetic field of a permanent cylindrical magnet positioned at the top of the spectacle frame, just above the affected eye. The attractive force of the system can be manually adjusted by reorienting the polar orientation of the frame magnet.

Importantly, this system is designed with a split configuration, meaning that there is no physical connection between the eyelid array and the frame magnet. The magnetic actuator approach, which has been successful in human clinical trials for unidirectional blepharoptosis, might be adapted for bidirectional eyelid paralysis by linking a servo motor to the frame magnet to produce polarity reversal. This approach is preferable to the use of an electromagnet which would generate heat and require a larger battery pack. We hypothesized that, by rotating this servo motor, the reversal of the static magnetic field could open and close the eye at a programmable rate, thereby achieving fully automated blinking in bidirectional paralysis.

The primary contributions of this article can be summarized as follows:

• First, a modification to the MLP design is proposed for its application in cases of bidirectional eyelid paralysis. This proposed design incorporates a servo motor, enabling the automatic reorientation of a cylindrical frame magnet. This adjustment allows the magnetic array to be attracted or repelled during the opening and closing of the eyelid.
• Second, a finite element analysis (FEA) for the device was conducted that provides insights into the eyelid-opening forces at various stages of the opening process. To validate the accuracy of this model, the results were compared with experimentally measured forces required for eyelid opening in a volunteer with bidirectional eyelid paralysis.
• Finally, as the theoretical capabilities of the design matched the experimentally obtained requirements, proof-of-concept testing of the proposed design was conducted on a volunteer, and the results are presented as evidence of feasibility.

## Methods

The proposed motorized MLP (MMLP) device consists of three main components:

1. Eyelid magnetic array.
2. Spectacle frame with neodymium–iron–boron (NdFeB) diametrically polarized cylindrical magnet (referred to as the frame magnet).
3. Open-loop control system consisting of a microservo motor, a microcontroller, user input potentiometers, and a power source.

The spectacle frame houses a servo motor attached to the frame at the base. The motor shaft houses a cylindrical NbFeB magnet of grade N52. The magnet diameter is either 9.5 mm or 12.7 mm, and the length is 12.7 mm. The poles of the magnet are oriented along the diametric axis of the magnet.

**Figure 1.**
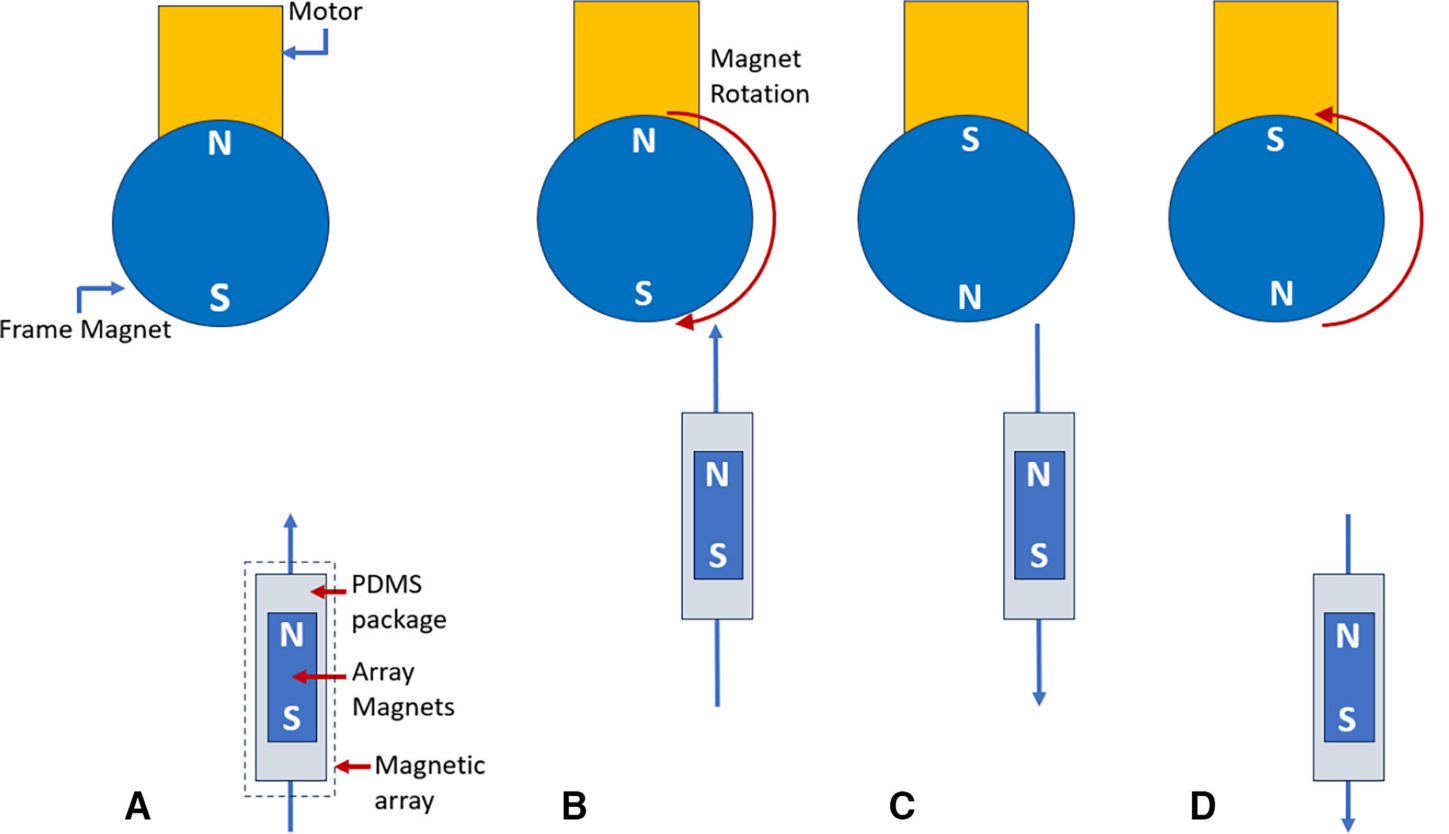
Simplified schematic diagram of MMLP design during the blink cycle. The *circle* represents the frame magnet, and the *rectangle* is the eyelid-attached magnetic array (eye and eyelid not illustrated). (**A**) When the eyelid begins to open, a low-magnitude attractive force acts on the magnetic array. (**B**) At the peak of opening, the array stops moving toward the frame magnet and is suspended in the fully open position due to resistance from the elastic properties of the eyelid skin or due to a physical barrier on the spectacle (not shown). Direct contact between the eyelid array and the frame magnet, which could be uncomfortable due to high forces, was prevented by an encasement of the frame magnet. The servo rotates the frame magnet to reverse force and initiate eyelid closing. (**C**) Frame magnet polar orientation during the closing of the eyelid. (**D**) The system was designed to produce a small magnitude of repulsion force at the point of full eyelid closure to allow for sustained eye closure. The motor rotates to start the cycle again. Components are not drawn to scale. The array is enlarged to make the details visible. Dynamic interactions during array movement, including magnetic flux torque, are not illustrated here. N, magnetic north; S, magnetic south.

The eyelid-attached magnetic array consists of three NbFeB permanent magnets enclosed in a PDMS biocompatible polymer (Fig. 1^2^A) using soft lithography techniques produced in our laboratory as described previously.^21^ The flexible PDMS array conforms to the natural shape of the eyelid. The magnetic array was attached to the eyelid by trimming Flexifix adhesive (Smith & Nephew, Watford, UK) to a size comparable to the upper lid. Part of the Tegaderm backing was preserved to aid in handling. The eyelid magnet size is 3 × 2 × 1 mm. The polar axis of the array magnets is along the 2-mm edge. The magnetic array can be easily placed on the eyelid by the user or by a caregiver using a self-applicator (Supplementary Fig. S4).^22^

**Figure 2.**
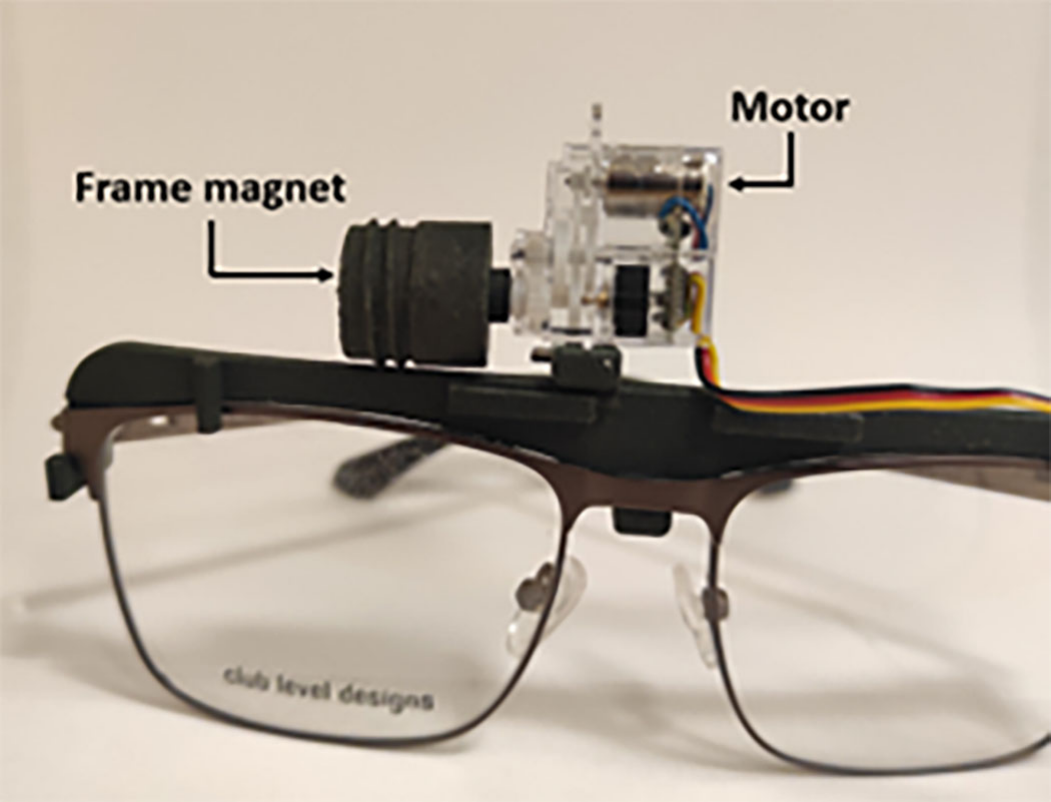
Spectacle frame of MMLP. The frame magnet is mounted on a rotation servo motor.

The servo motor is a Hitec HS-40 geared motor (Hitec RCD, San Diego, CA), shown in Figure 2, although many suitable and similarly inexpensive alternatives exist. The motor receives the control signal using three user-controlled potentiometers through an Arduino Nano (Arduino, Somerville, MA). The three potentiometers govern the angle traversed by the motor during each blink cycle, the speed of rotation, and the time delay between two blink cycles.

The functioning of the device can be understood by referring to Figure 1, which presents the design schematic of the device, as seen from the sagittal plane. During the majority of the cycle, the magnetic array is attracted toward the frame magnet, as the orientation of the magnetic poles of the frame magnet and array magnet enables attraction between them (Figs. 1A, 1B). After a customizable set time interval, the servo motor turns 180° and back. The time interval is set by the user for a comfortable experience. For a comfortable experience, the blink rate should match exactly with the unaffected eye. A time interval of 3 to 4 seconds can be selected to match the natural blink rate of 15 to 20 blinks per minute. During the maneuver, the magnetic orientation of the frame magnet attached to the motor is reversed, repelling the array magnet (Figs. 1B, 1C). Due to this repulsion, the array magnet closes the eyelid (Fig. 1D). When the motor returns to its initial orientation (Fig. 1D), the attractive force between the magnetic array and frame magnet restores, reopening the eyelid. If the rotation speed of the motor is high enough with no time lag between the forward and reverse rotation, the opening and closing of the eyelid can imitate a complete volitional blink.

**Figure 3.**
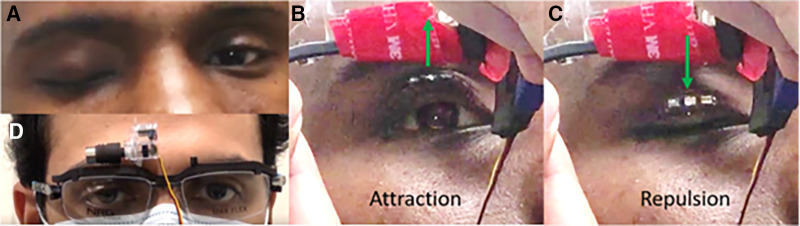
(**A**) Baseline condition of the subject with total and complete bidirectional right eyelid and facial paralysis. (**B**) The same participant in (**A**) is wearing the first prototype of an MMLP, held in place due to a lack of a frame/nose pad that fits appropriately. The fully paretic right eyelid is opened via magnetic force. (**C**) The paretic eyelid is closed via magnetic repulsive force after automated angular translation of the frame magnet driven by a servo motor (hidden behind the red material buffer) and Arduino controller. The eyelid movement was fully controlled with the prosthetic system (the participant had no motor function without the device). (**D**) Prototype 2 is fitted on a lab member without the red buffer for display purposes. In this design, the superior orbital fissure acts as a natural buffer, reducing the weight of the system.

It should be noted that the force magnitude on the magnetic array changes with the opening of the eyelid as the distance between the frame magnet and magnetic array changes. The attractive force will be minimal when the eyelid is entirely closed, gradually increasing as the eyelid opens and reaches maximum at the full opening of the eyelid. At this position, the magnet rotates, and a high repulsive force enacts the magnetic array. This repulsive force decreases as the eyelid closes and the distance between the frame magnet and array magnet increases. The cycle repeats when the motor rotates at the closed eyelid position, reenacting the attractive force on the magnetic array. Figures 3(b) and 3(c) shows the attraction repulsion phase respectively during device implementation. The hardware details for the system are given in Table 1.

**Table 1.** Motorized MLP Hardware Specifications


Motor	
Torque	0.75 kg/cm
Operating voltage	5 V
Dimensions	28 × 20.3 × 8.7 mm
Mass	7.44 g
Frame magnet	
Dimensions	12.7 × 12.7 mm
Material grade	N52
Mass	12.14 g
Array magnet	
Dimension	1 × 2 × 3 mm
Material grade	N52
Electronic components	
Operating voltage	5 V
Mass	51.47 g

The device underwent testing on a single volunteer who had complete and total bidirectional paralysis (no levator function, no observable orbicularis function). Written informed consent was obtained after explaining the risks and potential benefits of participation, following a protocol approved by the Mass General Brigham (formerly Partners Healthcare) Institutional Review Board. All procedures adhered to the tenets of the Declaration of Helsinki.

The volunteer was a 23-year-old African-American male with a remote history of severe traumatic brain injury, including a skull fracture, resulting in impingement of cranial nerves 3, 5, and 7 and, consequently, bidirectional eyelid paralysis. Remarkably, he reported no sustained cognitive or physical impairments related to his injury except for a chronically completely closed eye and facial palsy on the same side.

Manual measurements were conducted to determine the force required to open the eyelid. This involved tethering a FT03 force-displacement transducer (Grass Instrument Company, West Warick, RI) to the eyelid using a cotton thread and hydrocolloid skin adhesive. A Grass Instrument P122 AC/DC strain gauge amplifier manually recorded the final transducer readings.

The interpalpebral fissure (IPF) was also measured through image analysis using the MATLAB image processing library (MathWorks, Natick, MA). The process involved manually selecting points on the upper and lower eyelid for calculating the IPF. The distance between the points was calibrated from pixel to real space using an iris diameter equal to a population normative value of 11.7 mm, which was found in our prior work to be more accurate than a forehead marker.^18^ To account for the possibility of between-trial experimental errors based on the directional pull vector of the manually manipulated tether, the experiment was repeated multiple times with a constant value of measured force, as the analysis of the IPF opening occurred in the post-measurement phase.

FEA of the device was conducted to assess the attraction force exerted by the frame magnet on the magnetic array. The total attraction force was obtained through simulation at various IPFs. Assuming there was no force loss through the hydrocolloid adhesive, the frame magnet should spontaneously open the eyelid if the total attraction force exceeds the measured force required to open the eyelid, as recorded by the transducer.

The FEA encompassed the range from a complete eye closure IPF of 0 mm to full opening with an IPF of 9 mm (approximately 0.39 inches), with increments of 1 mm (about 0.04 inches). Due to the relatively low operating speed of the motor, the motion of the array was quasi-static. Hence, for this analysis, the effects of inertia were not considered. However, this was a conservative approximation, as any significant inertia in motion would aid the frame magnet when the eyelid moved.

**Figure 4.**
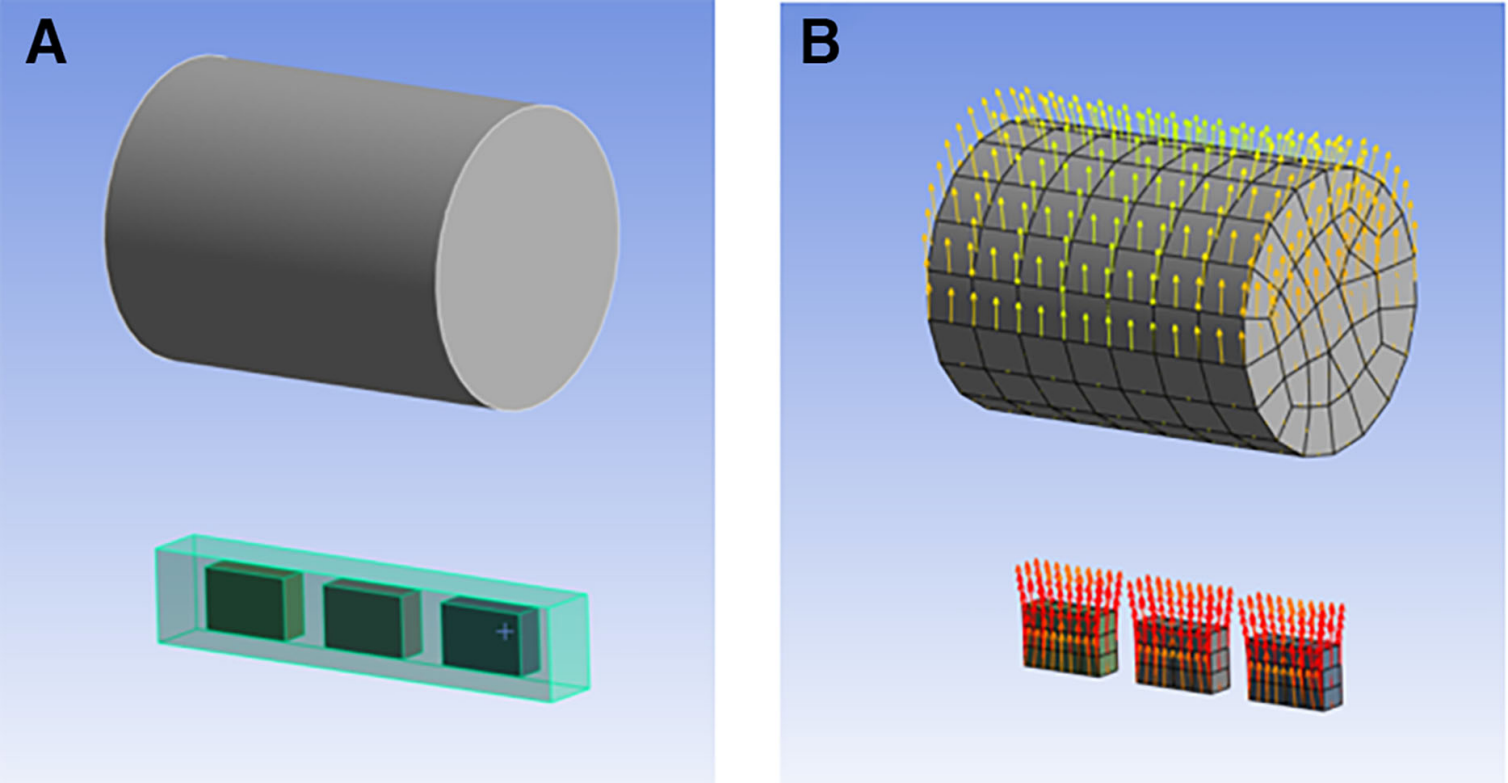
Finite element analysis of system. (**A**) The component arrangement of the system, including an upper frame magnet (12.7 × 12.7 mm) and three array magnets bound in a PDMS packing. (**B**) The magnetic field direction during the eyelid-opening phase.

The FEA was conducted using Ansys Student 2023R1. It involved modeling the frame magnet, array magnets, PDMS, and the surrounding air with a maximum of 107,049 nodes and 75,188 elements. The material properties used for the analysis are detailed in Table 2. Analyses were conducted at various IPF values by changing the relative distance between the frame magnet and magnetic array. Figure 4 shows the relative arrangement of bodies under analysis and the magnetic field direction used.

**Table 2.** Material Properties Used in Finite Element Analysis


Air	
Isotropic relative permeability	1.0
Neodymium iron boron (NbFeB–N52 grade)^23^	
Coercive force	12,300 Oersteds
Residual induction	14,500 gauss
PDMS^24^	
Isotropic relative permeability	1.0

## Results

**Figure 5.**
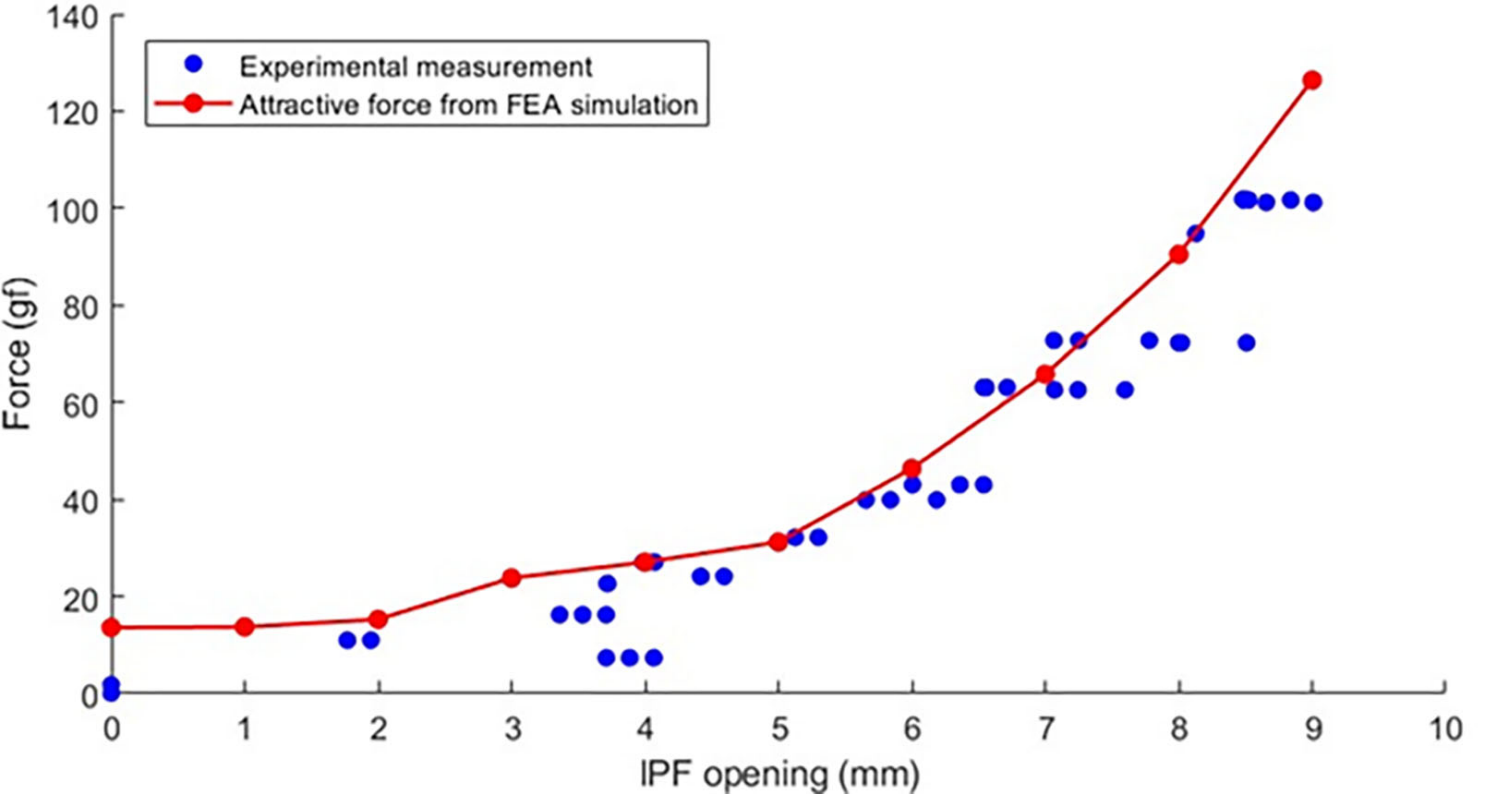
Variation trend of the attraction and repulsion force obtained through FEA with respect to the IPFs. *Red connected points* illustrate the trend in attraction forces obtained through simulation, juxtaposed with the measured forces (*blue data points*) at various IPFs. The figure shows that the attraction forces generated by the frame magnet closely align with the measured force levels in our volunteer.

The force necessary for initiating eye opening and sustaining various IPF openings as measured in our volunteer with the force transducer was consistent with the IPF opening forces derived from the FEA (Fig. 5). Therefore, it was anticipated that the device would elevate the eyelid effectively during the trial. For the subject under consideration, there was no difference between the opening and closing cycles as the patient was unable to generate any type of muscular force during the cycle.

Additionally, the average IPF for a specific measured force was observed, and the corresponding attraction forces from the FEA were tabulated (Table 3). In most cases, the force from the FEA matched or exceeded the measured force. Nevertheless, instances where the simulation force fell short of the measured force were evident (Fig. 5), which we attributed to variation in the IPF measurements caused by the challenge to maintain a perfectly vertical pulling direction during strain-gauge testing with the volunteer (the experimenter pulled upward manually). Regardless, the results suggest that the forces generated by the prototype system could be reasonably expected to elevate the eyelid during the attractive phase and depress the eyelid during the repulsive phase.

**Table 3.** Comparison of Experimentally Measured Force (Required Force) With Output of the FEA (Generated Force)

IPF (mm)	Measured Force (gf)	Opening Force from FEA (gf)
1.888	11.007	17.389
3.717	22.671	23.657
4.043	27.107	26.362
6.602	63.085	53.582
7.365	72.778	74.500
8.891	101.2	123.786

During the closing phase, the magnetic array is anticipated to experience a repulsive force of equivalent magnitude but opposite nature and is further assisted by gravity (requiring lower force); therefore, a (magnetostatic) analysis of the closing phase would be redundant and so is not presented. A system designed for the opening phase would be expected to generate more than enough force during the closing phase.

During the short trial of the designed prototype system with the volunteer, the results were as predicted by the FEA. The MMLP prototype effectively cleared the subject's visual axis (Fig. 3^4^,^5^B) and fully closed the eyelid, providing a complete volitional blink (Fig. 3C). For a video recording and comprehensive demonstration of the blink, please refer to Supplementary Video S1.

A frame-by-frame analysis of the trial video was performed to analyze the performance of the device. At the fully open position, the magnet successfully widened the IPF by an average of 8.52 mm, with a maximum of 10.03 mm. The magnet fully closed the eye with IPF minima of 0 mm during the closing phase of the cycle. The average time for motor half-rotation (i.e., polarity flip) was measured at 0.71 seconds, but the average time taken for opening or closing the eyelid was measured at 0.43 seconds, representing an average latency of 0.28 seconds from the start of the motor rotation to the start of eyelid motion, which might be attributed to the elastic structure of the eyelid and the inertia of the magnet. The participant rated their comfort during the trial as satisfactory and expressed willingness to consider wearing such a device if a wearable prototype were to be developed. There were no adverse events during the short ∼2-minute trial.

### Design Limitations and Future Improvements

The FEA was performed assuming quasi-static conditions. The latency between motor rotation and eyelid motion indicates that a dynamic model will be required for more accurate motion analysis and control. The eyelid motion lags the motor rotation, but the eyelid opens and closes fully. This suggests that the maximum eyelid speed is greater than that of the motor; therefore, a dynamic model-based controller is recommended for long-term applications to account for this lag. The controller is responsible for correctly tuning the angular velocity of the motor to maintain the desired blink time. Another method for controlling eyelid array behavior involves a detailed analysis of the polar orientation of the frame magnet and eyelid array magnet at various angular positions.^25^ The positioning of the frame magnet for fully open and fully closed positions can be adjusted to control torque on the eyelid array, which can be utilized to customize the appearance and function of the system. This can be a design constraint in addition to the opening force requirement.

In unilateral bidirectional ptosis, the initial plan does not entail the installation of a controller for synchronization but the timely initiation of blink. The patient will be trained to blink in coordination with the system to reduce complexity and lower power demands. Controllers, as described elsewhere,^26^^–^^28^ may be considered.

Spontaneous blinks can occur at a speed more than double the blink speed generated in this proof-of-concept study. Creating a spontaneous blink will not only require a faster rotation motion of the frame magnet but also a stronger frame magnet to overcome the elasticity and inertia of the eyelid under dynamic conditions. Rotation of such heavy magnets is a difficult task demanding torque levels that are orders of magnitude higher than what motors of a similar form factor can provide. One possible method of decreasing the time taken by the blink cycle is altering the motor rotation profile. Currently, a servo motor capable of rotation 180° is used. A DC motor, on the other hand, can rotate completely by 360°. Therefore, if a DC motor is used, then instead of stopping and reversing the rotation a complete rotation can be used to complete the blink cycle bypassing the need for a complete stop. This can decrease the blink cycle time without significantly increasing the power requirements. In bilateral cases, achieving blink synchronization is feasible, as this system will involve two motors, one for each eye, both of which can be triggered from a single controller, irrespective of the type of motor used.

Additional information about the dynamic properties of the eyelid and its motion, long-term corneal health, and cosmetic appearance will be required to obtain an acceptable control profile. Such data can be recorded from the general population and used to generate the control profile for a particular patient with bidirectional ptosis. Such an experiment should be conducted in the future as the development of this device progresses. The force between the magnets can be adjusted by changing the size of magnets (frame magnet or array magnets, or both), changing the number of array magnets, and changing the distance between magnets. These parameters can be considered for a potential customized solution for patients.

Several other potential barriers are anticipated in the current design, including concerns related to frame stability, unacceptable amounts of spectacle frame weight, and power requirements for proper functioning during extended use. Frame stability, particularly under dynamic actions such as magnet rotation, is a valid concern due to the relatively high mass density of the NdFeB magnet employed. The entire frame might become unstable due to the rotation of an unbalanced mass. This situation may arise from manufacturing defects, misalignment of the magnet and motor shaft, or improper motor placement. One possible measure to enhance stability involves the utilization of patient-specific custom three-dimensional printed frames, a strategy previously employed for standard MLP designs with success.^20^ Considering the patient's anatomy, support points can be preferentially placed near the frame magnet, increasing stability. Additional support structures can be incorporated while ensuring comfort.

The power source significantly influences concerns regarding weight and power requirements. To address this, a lightweight lithium-ion battery can be positioned on the opposite side of the affected eye to counterbalance the static moment induced by the motor and magnet. The current drawn by the motor was measured using an ASC712ELCTR-30A-T sensor (Allegro MicroSystems, Manchester, NH). The motor draws a current of 37.88 milliampere (mA) when using the 12.7-mm magnet. Given the operating voltage of 5 volts (V), the motor consumes power of nearly 0.19 watts (W). Given the small current requirements, small batteries can be mounted on the frame. Small batteries with a capacity of 400 milliampere-hour (mAh), giving a running time of over 10 hours, can have a footprint of nearly 1 × 1.5 inches and therefore can be a viable option to be fitted on the frame.

Another feasible option is to power the device using a phone or a power bank via a USB cable. This will provide a more reliable and convenient option for the end user but will not provide the balancing effects of a battery mounted on the frame. A test and redesign bench-to-bedside process involving an engineering team, clinicians, and patients will be essential to acquire the experience and knowledge necessary to achieve clinically acceptable success rates.

## Discussion

The MMLP device, introduced here for the first time, is presented as an auspicious approach for reanimating the eyelid in bidirectional eyelid paralysis, achieving a fully automated and complete opening and closure during the blink. To our knowledge, this is the first proof of concept in a living patient for any prosthetic technology to address bidirectional paralysis in or around the eye and face. Prior work with magnetic actuators for unidirectional severe blepharoptosis has already demonstrated, via open-label^19^^,^^20^^,^^25^ and a randomized clinical trial,^21^ high fitting success rates in more than 50 participants with severe ptosis with very few adverse events in the short term^29^ or longer term.^22^ Hence, it was expected that the MMLP would achieve similar success in opening the eye in bidirectional paralysis, as demonstrated in this specific case. The results by FEA predicted that the necessary forces could be achieved with a 12.7 × 12.7 N52 diametric frame magnet and a magnetic array with array magnets, which was subsequently confirmed in vivo. The response observed in this pioneering case was excellent, with the expected full eyelid elevation in the opening phase as well as rapid and complete fully automated blinking during the closing phase. The response was consistent over several cycles during the ∼1 minute trial. Video analysis of eyelid motion hints that the MMLP generates a blink speed that approximates the speed of a volitional blink and approaches the spontaneous blink velocity.^30^

The size of the system was considered clinically feasible, based on the clinician author (K.E.H.) and participant feedback and was supported by the participant's strong interest in wearing the device for an extended trial after experiencing its impact on their eyelid function. Additionally, the comfort level was found to be acceptable by the participant during this brief trial. A larger human subject study with longer wear times is appropriate at this stage. As this patient also has a cranial nerve V palsy, the sensation on their eye, orbit, and face may be diminished, and this limited sensation may have contributed to the tolerability of the device. It must be noted that, with our experience with the traditional MLP, discomfort does not arise from the cornea but is related to pulling sensations on the eyelids. The motor noise is not too high but it might not be possible to neglect this level of noise when used for a long time. The noise is attributed to the gear train attached to the motor. Redesigning the system to avoid the need for a gear train will significantly reduce the noise.

Safety concerns that could result from the magnetic interaction of the device with other common items such as electronics, magnetic resonance imaging, orbital and facial reconstruction hardware, or implantable cardioverter–defibrillators (ICDs) have been considered and are being monitored for issues in prior and ongoing chronic use studies. The safety concern is bidirectional; that is, the safety concern can arise from an external magnetic field that is applied on the device or the magnetic field from the device acting on other components. For the former case, the concerns are minimal because of a variety of factors. First, the device is fully external so in case of emergency the device can be easily removed. Second, the biggest interaction with any external field will be that by the frame magnet, as it is the strongest magnetic element. The frame is not attached and will fall off the user if it interacts with an external field. The array magnets, though attached with Tegaderm tape, are enclosed in a PDMS packing. In our experience, this PDMS packing is easy to break, and array magnets under the external field will break out of this packaging before causing any serious injury to the skin due to a pulling action. The users should be informed of this concern and should be advised to refrain from using the device in regions of a high magnetic field such as magnetic resonance imaging rooms and near large magnets. The functionality of MLPs is susceptible to other magnetic fields but such effects can safely be assumed to be minimal because the region of operation of array magnets is greatly dominated by the field from the frame magnet on account of its proximity and strength.

A second type of magnetic interaction must also be discussed—the influence on other devices due to the magnetic field from the device. Modern orbitofacial reconstruction implants are mostly made of titanium, which is paramagnetic. Electrical implants such as ICDs can theoretically be affected by small neodymium magnets,^31^^,^^32^ but there are no reported cases in the clinical literature. Users with ICDs are advised to prevent MLP contact with the chest and regions adjoining the ICD site as a precautionary measure. Many recent ICDs are rated MRI-safe. Such ICDs are practically immune to interaction with any external magnetic field.

It was discovered in this experiment that the flange thickness (the bonded section between the PDMS cube containing the three cube magnets that connect it to the skin adhesive outside surface) may be an important parameter for device functioning. During frame magnet rotation, the array has the propensity to roll in addition to the expected primary translational movement.^25^ The moment of inertia of the array seemed to be increased by an expanded flange area and thickness, thereby improving its resistance to rotation. Furthermore, a wider flange can function as a physical barrier against rotation due to the limited available space. Further studies might also examine torque on magnetic array in addition to force.

Although bidirectional eyelid paralysis is rare, it has dire consequences and deserves the attention of the ophthalmic community. Research on this condition faces challenges in recruitment due to its rarity and the absence of a unique International Classification of Diseases, 10th Revision (ICD-10) code. The establishment of a registry is a potential solution. It could provide an avenue for recruitment, capture data on prevalence and quality-of-life impacts, and provide a platform for education, support, and public awareness.

The MMLP approach offers several advantages over the extension of other methods,^6^^–^^17^ including solenoid, mechanical, soft ptosis crutch/speculum robot, or electromagnet approaches. First, MMLP allows for complete non-invasive testing, bypassing the need for animal studies and enabling direct testing in humans. In contrast, the proposed solenoid tether system has not been suggested as an externally fixed device. It would be challenging to prevent the tethers from slipping under hydrocolloid adhesive, even with surgical implantation, and the lateral force generated by the tether, although somewhat comparable to forces generated by the orbicularis, differs significantly from the upward and posterior force generated by the levator. This may result in an unnatural appearance and repeated mechanical pressure on the corneal apex, which can be problematic for patients with fragile corneas. Although the MMLP also exerts some mechanical pressure on the corneal apex, it does so to a much lesser extent, as no lateral tension is involved. In the case of the soft crutch system,^33^ it is likely to share similar drawbacks, including the risk of repeated mechanical pressure and cosmetic unacceptability, as the prototype appears bulky in photographs. Notably, our participant expressed enthusiasm at the prospect of having a system to take home for a more extended trial. A more optimized design will be developed in the future to address these issues.

The optimization of the level of control granted to the subject is deemed crucial. Although providing the subject with some control over the device is advantageous, there should be limits on how much the calibration can be adjusted for safety reasons. The subjects can be allowed to activate and deactivate the device to synchronize blink initiation manually or to control the frequency of blinking within limits. During the design stage, the blink cycle and rate duration should be precalibrated, and blink synchronization should be accomplished through subject training before utilizing the frame.

The feasibility of delivering sufficient forces through the designed system has been demonstrated. Due to the unique capabilities of this device, it is anticipated to exert a significant impact and prove highly beneficial to patients afflicted by such a condition. The theoretical analysis conducted using FEA closely aligns with the measured requirements, indicating a sufficiently intricate analysis. This alignment is further reinforced by the satisfactory performance of the device during the trial. Therefore, it is believed by the authors that this analysis is adequate for establishing the proof of concept. The device will be tested on multiple subjects to quantify its performance and capabilities in future endeavors.

## Supplementary Material

Supplement 1

Supplement 2
