# Supplementary material for: A Magnetic Actuator Device for Fully Automated Blinking in Total Bidirectional Eyelid Paralysis: First Proof of Concept in a Human Participant
Source: Transl Vis Sci Technol. 2024 May 2;13(5):2. doi: 10.1167/tvst.13.5.2 (PMC11077920; doi:10.1167/tvst.13.5.2)
Supplement: Supplement 1 [file tvst-13-5-2_s001.pdf]

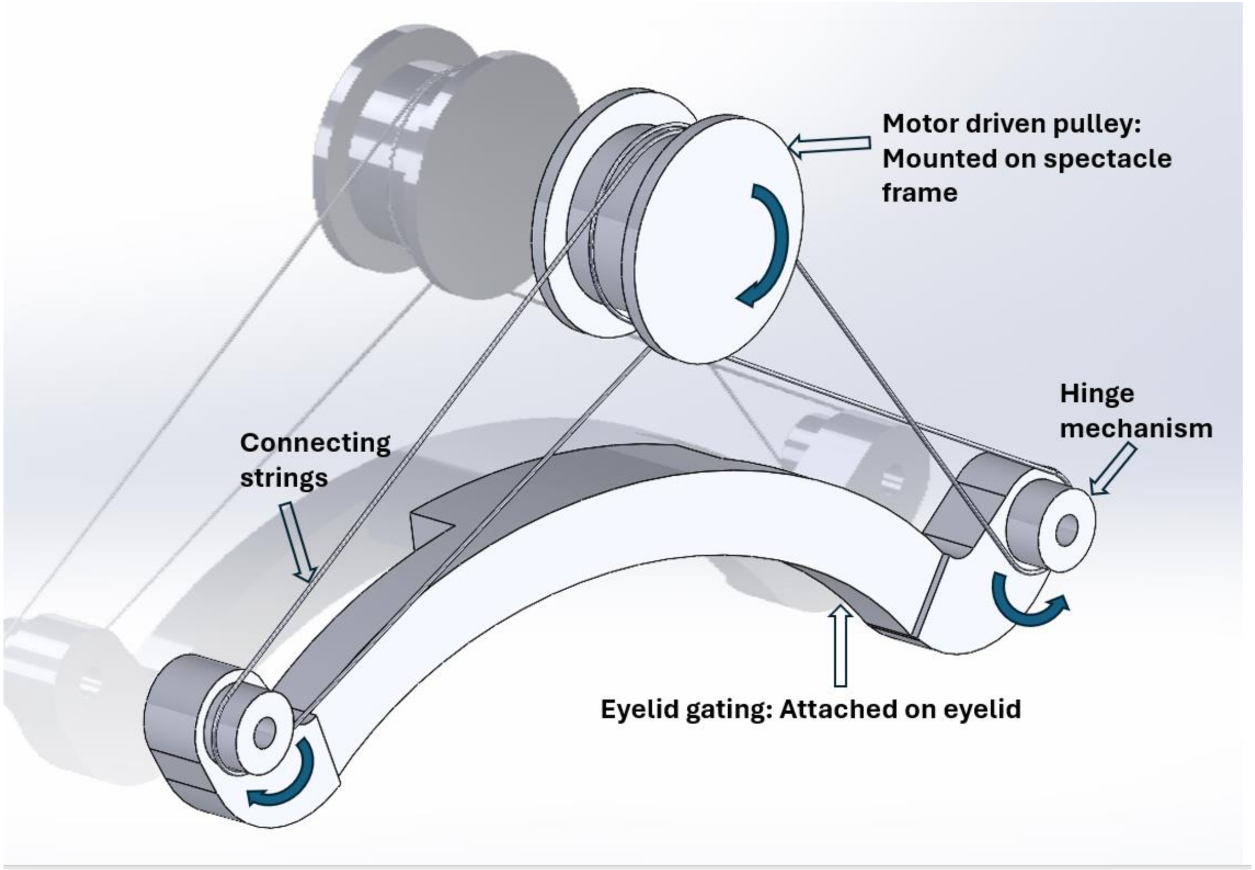

Supplementary Figure S2: Concept sketch of eyelid gating mechanism [10-12]

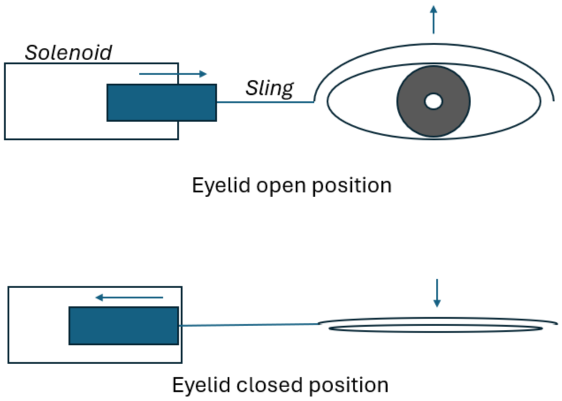

Supplementary Figure S3: Concept sketch of solenoid lid implant [13 – 17]

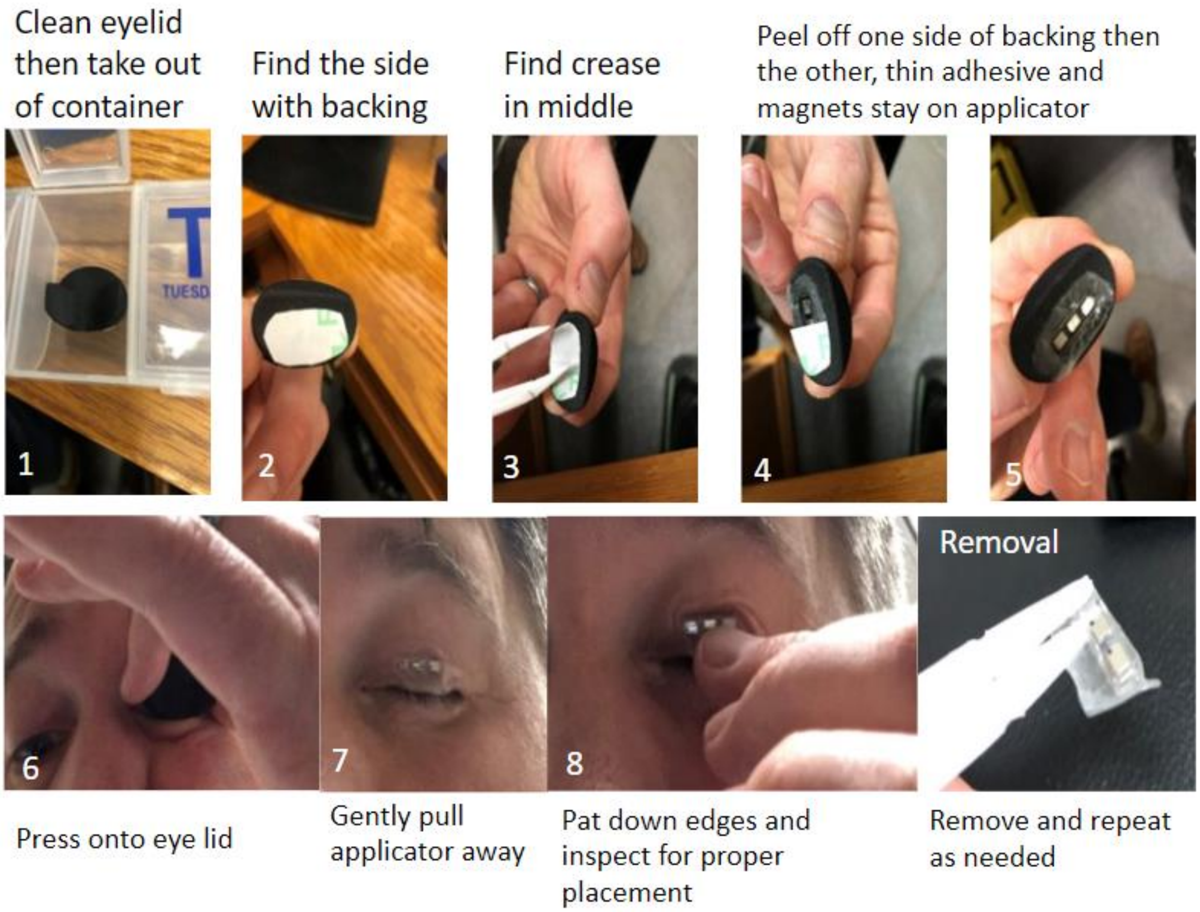

Supplementary Figure S4: Array self applicator.
